# Physiological Evaluation of Quarter Horses Undergoing Five-Month Taming Focused on Hippotherapy

**DOI:** 10.3390/ani16131980

**Published:** 2026-06-26

**Authors:** Lara C. S. Costa, Emmanuel Arnhold, Jorge D. Passamani, Alexandre R. A. Cardoso, Letícia C. Celeste, Kate M. C. Barcelos

**Affiliations:** 1 School of Veterinary Medicine and Zootechnics (EVZ), Federal University of Goiás (UFG), Goiânia 74690-900, Brazil; laracarolynne@discente.ufg.br (L.C.S.C.); emmanuelarnhold@ufg.br (E.A.); 2 National Association of Equine-Therapy Services (ANDE), Brasília 70636-000, Brazil; pahais@hotmail.com (J.D.P.); alexandre.racardoso@gmail.com (A.R.A.C.); 3 Fonoaudiologia—Speech-Language Pathology, University of Brasília (UnB), Brasília 70910-900, Brazil; leticiaceleste@unb.br

**Keywords:** equine, heart rate, cortisol, thermography, hippotherapy, horsemanship

## Abstract

Preparing horses for hippotherapy programs requires management approaches that prioritize animal welfare and facilitate adaptation to novel stimuli. This study monitored physiological responses during the implementation of a progressive taming protocol for Quarter Horses intended for hippotherapy. Seven animals were monitored over a five-month period, including an acclimation phase and three taming stages. Continuous heart rate, plasma cortisol concentration, and ocular infrared thermography were assessed before and after exercise. Baseline (*p* = 0.0466) and minimum (*p* = 0.0095) heart rate values decreased over time, suggesting progressive physiological adaptation. Maximum heart rate (HR_Max_) was higher during the initial phases, coinciding with the introduction of novel stimuli, and decreased in subsequent months (*p* = 0.0272). Lacrimal caruncle temperature increased at the beginning of taming (*p* = 0.002), followed by stabilization throughout the experimental period. Plasma cortisol concentrations did not differ between sampling times or among months (*p* ≥ 0.05). Taken together, the physiological indicators evaluated suggest that the progressive taming protocol supported gradual physiological adaptation throughout the taming period. These findings indicate that a progressive approach may be useful for preparing horses intended for hippotherapy, although additional studies incorporating behavioral and welfare assessments are warranted.

## Simple Summary

Horses used in hippotherapy must be calm, predictable, and able to cope with a variety of environmental, tactile, auditory, and visual stimuli. However, standardized protocols specifically designed to prepare horses for this activity remain limited. Therefore, this study monitored physiological responses during the implementation of a progressive taming protocol for Quarter Horses intended for hippotherapy. Heart rate, ocular infrared thermography, and plasma cortisol concentrations were evaluated in seven horses over a five-month period. The results showed a gradual reduction and stabilization of heart rate and ocular temperature parameters throughout taming, while plasma cortisol concentrations remained stable over time. Taken together, these findings suggest that a progressive taming approach may support physiological adaptation in horses prepared for hippotherapy.

## 1. Introduction

Historically, horses have been used in various contexts, including for transportation, warfare, sports, leisure, and therapeutic activities. Reports of the therapeutic use of horses date back to antiquity, when Hippocrates described their use as a healing aid between 458 and 377 BC. Furthermore, across different eras, particularly after World War I, therapeutic practices became more consolidated [1,2,3,4]. This has increasingly been integrated into One Health concepts regarding human–animal interactions to promote welfare and quality of life [5].

In this context, animal-assisted interventions (AAIs) include therapeutic and educational practices involving animals within health and rehabilitation settings. In the equine sector, terms such as equine-assisted interventions (EAIs), equine-assisted therapy, hippotherapy, and equine therapy are similar but are frequently used with differing ideological implications depending on therapeutic goals and regulatory standards [6]. In Brazil, the term Equoterapia^®^, designated and owned by the National Association of Equine Therapy Services (ANDE-BRASIL), was registered in 1989 to encompass different horse-assisted modalities, including hippotherapy, education/re-education, pre-sport practice, and para-equestrian sports activities [3,7], each with a specific therapeutic or social inclusion purpose.

Equotherapy^®^ was officially recognized in Brazil as a therapeutic approach by the Federal Council of Medicine (CFM) in 1997 and by the Federal Council of Physical Therapy and Occupational Therapy (COFFITO) in 2008 [8,9]. In 2019, it was codified into law as a rehabilitation method for people with disabilities (Law No. 13.830/19) [7]. Notably, Brazilian legislation and professional statutes recognize and adopt the term Equoterapia^®^ for horse-assisted rehabilitation, using it as the standard terminology in the country [7]. However, in this study, the term “hippotherapy” was used throughout the manuscript strictly in accordance with its international definition, specifically referring to the health-related rehabilitation modality in which the horse serves as a kinesiotherapeutic instrument [3,10].

The efficacy and safety of hippotherapy depend directly on the use of well-trained horses that exhibit predictable behavior, tolerate diverse stimuli, and adapt well to the therapeutic environment [3,11]. Thus, the selection of horses for this purpose can begin by evaluating characteristics such as temperament, gait consistency, responsiveness, and behavioral stability, which are essential prerequisites for this activity [12,13,14].

In Brazil, the Quarter Horse (QH) breed is widely used for this service due to its high availability in the market, as it is among the largest equine populations bred in the country. A docile and intelligent temperament, along with ease of handling, is an important characteristic when choosing an animal for interventions involving people with disabilities (PWD). Furthermore, the wide range of QH lineages adapted to the breed’s most diverse sports modalities allows for more detailed selection of specific traits, such as weight, withers height, and even coat color, thereby offering a wide range of animal options. Biomechanically, these animals exhibit the three natural gaits (walk, trot, and canter) required for broader riding practices and have demonstrated good walk regularity [15], an essential characteristic for use in equine therapy.

Despite these selection criteria, introduction to an Equine-Assisted Services (EAS) environment exposes horses to novel stimuli, equipment, and management paradigms that represent distinct psychophysiological challenges for an inexperienced horse [16,17]. In equitation science, preparing horses for these demands is based on learning theory, primarily habituation and systematic desensitization, to mitigate innate fear and avoidance responses [11,18,19]. Historically, conventional or regional training approaches sometimes resorted to high-pressure tactics, excessive and misapplied negative reinforcement, or punishment, which could cause acute stress or chronic behavioral frustration [16,18]. On the other hand, structured, animal welfare-oriented training methodologies prioritize gradual handling, clear signaling, and low-stress habituation to promote stable behavioral adaptation without compromising equine welfare [20,21,22].

To objectively monitor equine coping mechanisms during these preparatory phases, physiological assessments are necessary. Neuroendocrine responses are commonly tracked through changes in glucocorticoids; however, rather than serving solely as an index of psychological “stress”, cortisol is fundamentally involved in energy homeostasis, metabolic substrate mobilization, and the regulation of behavioral arousal [23,24,25] and therefore can serve as an excellent physiological marker. In both modern exercise physiology and animal welfare analyses, longitudinal monitoring of plasma cortisol concentration can reveal the adaptive stage to training over time, as well as specific workloads or consistent cognitive changes [26,27]. Similarly, measuring HR in horses is a well-established, non-invasive method that has been used for years to provide information on the activity of the animal’s autonomic nervous system [28]. Changes in cardiovascular parameters are expected when working with horses in different training modalities. However, abrupt changes in HR in response to novel stimuli may indicate discomfort and increased physiological stress, directly influencing training quality and the behavioral responses of various horses [29,30].

Infrared thermography (IRT) is scientifically validated and non-invasive [31,32,33], and is gaining prominence in animal welfare assessments because it is directly related to core body temperature. One of its most recent applications is ocular IRT, which has been increasingly used to identify physiological and behavioral changes associated with emotional stress, thereby aiding the monitoring of health, performance, longevity, and quality of life in horses. The absence of direct physical contact with the animal makes this technique highly suitable for welfare assessments, and its rapid image acquisition further supports its use in field conditions during training and management [31,32,33].

Thus, this study aimed to physiologically evaluate (HR, plasma cortisol, and ocular IRT) a taming protocol for therapeutic riding in Quarter Horses over 5 months of taming.

## 2. Materials and Methods

This study was approved by the Animal Use Ethics Committee of the Federal University of Goiás (CEUA-UFG), Brazil, under protocol no. 011/24. All animals underwent a complete clinical examination, including rectal temperature assessment, as well as thermographic and hematological evaluations performed by a qualified veterinarian to ensure their adequate health status before the beginning of the taming protocol and during the acclimatization period.

Animals: The project selected seven QHs, duly registered with ABQM, for their docile temperament and three well-marked gaits: walk, trot, and gallop [15]. All the animals were geldings over 3 years old (5.43 ± 0.53 years), with an average withers height ranging from 1.31 m to 1.42 m (1.38 ± 0.05 m; measured via standard hypsometer), and a body weight between 310 and 415 kg (354 ± 34 kg). Their age was considered compatible with the young adult phase, when horses have sufficient physical maturity for mounted work, as they generally begin training at 2–3 years old [6]. The selection also considered morphological characteristics compatible with use in hippotherapy, prioritizing animals with medium conformation and a lower stature to facilitate the physical support and ergonomic reach of the side-walking therapists [3]. To meet these requirements, animals from a working or reining lineage were selected, as this genetic line is phenotypically characterized by a more compact, shorter, and stockier biotype. Prior to the experiment, the horses were entirely unbacked and untrained, having only experienced basic halter management. The animals were transported from local ranches (with a transit duration of 4–12 h) and underwent a strict 30-day acclimatization period. All individuals were behaviorally healthy, showing no history or clinical signs of stereotypic behaviors or abnormal reactivity outliers.

The QHs were acquired from farms in the region, where they lived on pasture, were already accustomed to halter handling for general hygiene (grooming) and hoof trimming, and were supplemented with concentrated feed and mineral salt. They were transported in horse-specific transport to the ANDE-BRASIL facilities at an equestrian center in Granja do Torto, Federal District, Brasília, Brazil, where they remained throughout the experiment. The animals were kept in individual, well-ventilated 4 × 4 m masonry stalls, where the horses could see the other animals from the front of the stall and through the door. The stalls had a concrete floor with a drainage grate, wood-shavings bedding, a masonry trough, and an automatic plastic waterer with an individual covered float. Water was provided ad libitum. The diet corresponded to 2.25% of each animal’s body weight individually in % dry matter (DM), consisting of concentrate (commercial balanced feed) and roughage (Tifton hay) in a 30:70 ratio. The concentrate was divided and administered twice daily (7 a.m. and 4 p.m.). The hay was divided into portions of similar weight and provided three times a day (morning, afternoon, and evening). Mineral salt was provided in a separate trough ad libitum. All animals remained in the stalls until the start of collection, release into paddocks, and taming activities.

Experimental Design: The study spanned 5 months (November to March), with each animal assessed monthly. Data were collected before (baseline), during, and after taming activities, according to the variable analyzed (HR, plasma cortisol, and ocular IRT). November served as the baseline control month during the animals’ initial adaptation period, so only resting baseline data were collected. From December onward, the Progressive Taming Protocol for Hippotherapy (PTPH) was implemented and evaluated monthly during its active taming phases.

HR: It was continuously assessed using a Polar H10 device (Polar, São Paulo, SP, Brazil) positioned around the horse’s thoracic region. This is a lightweight (21 g), compact (34 × 65 × 10 mm) HR monitor with an adjustable elastic strap made of comfortable textile material, using a CR2025 battery that lasts up to 400 h. The sensor was connected to the device to monitor beats per minute (HR) via Bluetooth Low Energy, ANT+ through a coded 5 kHz transmission. HR was recorded using the Polar Equine program on a Samsung S7 tablet. The assessment obtained the following parameters per month of taming: baseline HR (HR_0_), minimum HR (HR_Min_), average HR (HR_Ave_), maximum HR (HR_Max_), and final HR (HR_Fin_). Thus, it was possible to evaluate variations in physiological stress indicators throughout the taming process, allowing inferences about the animals’ adaptation to the proposed protocol. Data were collected once a month after randomly selecting the animals, with the animals distributed over two consecutive evaluation days (four on the first day and three on the second). HR_0_ was measured in the stable, starting at 8 a.m., before the start of activities. Subsequent collections were carried out before, during, and immediately after taming. A taming session compatible with the taming level of each taming stage was simulated on the evaluation day. Activities consisted of leading and groundwork exercises in Phases 1 and 2 and a taming circuit in Phase 3, initially leading the animal on a lead and then riding it.

**Figure 1:**
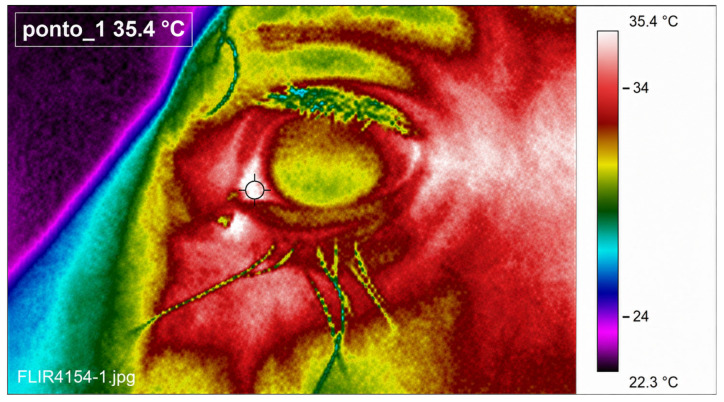
Thermogram of the region of interest (lacrimal caruncle). Thermogram showing higher thermal activity in the ocular region, indicated by the highest-temperature area (black/circled) in the lacrimal caruncle of the left eye of a Quarter Horse (QM). Image captured at a distance of 0.50 m, with the eye and the infrared thermography (IRT) camera aligned perpendicularly, using the Rainbow HD color palette.

Ocular IRT: The capture distance was standardized at 0.5 m from the animal’s left eye, directed to the lacrimal caruncle (Figure 1). The equipment was positioned at 90° to the sagittal plane, as measured with a digital laser tape measure [34].

Ocular IRT images were captured using a FLIR E40 camera (FLIR Systems, Wilsonville, OR, USA), featuring a focal plane array resolution of 160 × 120 pixels and operating within a temperature range of −20 °C to 650 °C, with a manufacturer-reported absolute accuracy of ±2 °C or ±2%. The camera presented a thermal sensitivity of <0.07 °C at 30 °C. For each session, three consecutive images were obtained per eye, and the image with the highest focus and the fewest motion artifacts was selected for analysis. Images were stored using randomized numeric codes to blind the analysis regarding animal identity, date, and taming phase. Thermal analysis was subsequently performed by an independent evaluator using FLIR IRISoft software (1.0.11.2). During the experiment, the surface emissivity was fixed at ε = 0.98, which is appropriate for mammalian skin [33]. To minimize environmental confounding factors, the ambient temperature (21.7 ± 1.3 °C) and relative humidity (75.2 ± 10.4%) inside the pavilion were monitored daily using a digital thermo-hygrometer and the readings were entered into the camera calibration settings prior to image acquisition. Regional weather data obtained from Apple Weather^®^ (iOS) were used only for environmental characterization.

On each taming day, the first collection began at 8:30 a.m. in the stable, sheltered from direct light, with baseline thermographic images captured 0.5 m from the left eye [34] before exercise. The Images were evaluated immediately after capture to avoid errors and artifacts (blur, flies, dirt, closed eye), and the capture was repeated if necessary to ensure its quality for analysis. Following the activity, the second collection took place the same day, in the covered round pen or arena, immediately after the exercise session. The collection and work sequence were randomized by drawing, and the animals were evaluated on two consecutive mornings (four animals on the first day and three on the second day). The images were stored on a 128 GB SD memory card for later processing on a computer using IRISoft software (PoliScan^®^, FLIR Systems Inc., Wilsonville, OR, USA), selecting only the images that presented the best technical quality, with a sharp focus, the absence of thermal artifacts, and the eye fully open.

Plasma cortisol: Blood samples were obtained via jugular vein venipuncture to measure plasma cortisol levels [35]. The site was previously cleaned; the samples were collected using disposable needles (25.0 × 0.8 mm) and stored in 10 mL BD Vacutainer^®^ tubes (heparinized) for subsequent processing. They were collected from 10 a.m. onwards, considering the circadian rhythm of cortisol in horses [36] and the morning cortisol peak (between 6 and 8 a.m.) [37], to standardize the tests and avoid confounding effects. The collection and work sequence were randomized by drawing, with evaluations conducted on two consecutive mornings (four animals on the first day and three on the second). Each horse underwent collections at two different times: first, pre-exercise samples (10 a.m.) were collected inside the stables (5 to 10 min between entering and leaving the stable), thus obtaining baseline plasma cortisol samples; then, samples were collected in the round pen or arena (both covered), immediately after completing the proposed exercises, to compare any changes resulting from the taming. All samples were transported under refrigeration to the Veterinary Multipurpose Laboratory of the University of Brasília (UnB, Brasília, Brazil), where they were stored. At this stage, the samples obtained in heparinized tubes were centrifuged for 10 min (Kasvi^®^, model K14-4000, Pinhais, PR, Brazil) at 4000 rpm to separate plasma. Then, the refrigerated plasma was sent to the specialized Veterinary Endocrinology Laboratory at BET Labs (Niterói, RJ, Brazil) for the measurement of blood cortisol levels. The technique used was solid-phase radioimmunoassay (RIA) with commercial kits (Siemens Healthcare Diagnostics Inc., Tarrytown, NY, USA). All tests were performed in duplicate.

The animals underwent a specialized Progressive Taming Program for Hippotherapy (PTPH). Unlike traditional equestrian pathways, where horses must first undergo years of general riding school or sport disciplines before being adapted to therapeutic work, this protocol was designed to transition completely naive, unhandled horses directly into safe therapy animals. The core value of this specialized approach lies in combining intensive behavioral desensitization with an integrated functional gymnastics program. This dual strategy ensures that the necessary physical conditioning and basic equestrian movement awareness develop in parallel with therapeutic habituation, qualifying the horse directly for hippotherapy services upon program completion.

PTPH Steps: 1. Adaptation—dedicated to environmental acclimation, facility familiarization, and the establishment of baseline resting physiological control profiles (recommended: 30–45 days); 2. Phase 1—Communication and Integration (recommended: 30–45 days); 3. Phase 2—Desensitization (recommended: 30–45 days); and 4. Phase 3—Capacity Building and Skills Development (recommended: 90–150 days). A gymnastics program was also conducted throughout the entire experimental period. It consisted of physical conditioning exercises, functional gymnastics, and basic training, carried out continuously (recommended: throughout Phases 1, 2, and 3 on alternate days to those with the other exercises approached in the other specific phases).

General recommendations for the taming period: The proposed durations for each taming stage are based on the protocol routinely used by the ANDE-BRASIL team to prepare horses for hippotherapy. Because the taming is individualized, the progression time between stages may vary among animals, influenced by factors such as previous experience, breed, and temperament. The stages were standardized to the minimum time necessary to obtain consistent results and reduce experimental biases, solely for comparison between individuals. The values adopted at each stage in this research are described in detail in the following sections. The experiment lasted 5 months, including 30 days of adaptation (during which no data were collected on the analyzed variables), followed by 4 months of the taming protocol and experimental evaluations. The PTPH consisted of the following stages: Adaptation in November (30 days); Phase 1—Communication and Integration (30 days); Phase 2—Desensitization (30 days); and Phase 3—Capacity Building and Skills Development (60 days), concluding in March. The gymnastics phase was performed continuously throughout Phases 1, 2, and 3.

Acclimation Phase—Familiarization and Coexistence (30 days): This period began with the animals’ arrival at the ANDE-BRASIL facilities and included clinical and laboratory examinations, preventive dental treatments, vaccination, and deworming to ensure the health of the animals included in the experiment. The adaptation to the new environment then proceeded over 30 days. During this period, the animals underwent activities to familiarize them with the stalls, daily handling, and the routine of a hippotherapy center. These activities included individual handling, hygiene, manipulation, halter training, and release into collective paddocks, all aimed at familiarizing them with the environment and routine practices. A single person performed daily handling at all study stages, leading the animals in the stalls and handling them generally, under the trainer’s supervision. During the taming phases, a single trainer led all animals throughout the experimental period to minimize variations associated with human handling. HR, ocular IRT, and plasma cortisol levels were measured in the stalls, with a single baseline sample obtained from each animal during this period.

- Acclimation Phase—Steps:

Management and Health: Complete veterinary clinical examination (including baseline rectal temperature measurement), full blood count (hemogram and leukogram profiling), deworming, vaccination, and basic dental management.

Activities: General daily ranch management and basic halter handling only.

BPM (beats per minute): Assessment of resting heart rate inside the animals’ individual stalls to establish baseline resting cardiac parameters.

Plasma Cortisol: Collection of a single baseline blood sample per animal inside the stall to determine initial resting cortisol levels.

Ocular IRT: Ocular infrared thermography assessment conducted strictly inside the initial stall environment to capture baseline resting eye temperature.

Phase 1—Communication and Integration (30 days): This stage began with groundwork in a round pen, using non-riding activities to develop communication between the trainer and horse, and to promote the animal’s body awareness and physical preparation. This approach aims to facilitate future adaptation to riding, since the horse already understands the basic commands executed on the ground [38]. Phases 1 and 2 were conducted progressively, following a structured sequence of ground exercises. The equipment included a halter, long lead, vocal commands, body language, a stick, and a flag stick (training flag).

The animals were led to the round pen by the trainer using a halter and long lead, with both initially positioned in the center of the area. To begin the exercise, the trainer indicated the direction of movement with one hand and, with the other, moved the stick posteriorly toward the croup area without direct physical contact, thereby stimulating the animal’s movement using the principles of pressure and release [11]. The exercise consisted of circular movements to the right and left, with five repetitions for each side. At the end of each turn, a vocal command to stop was given, followed by the animal approaching the center of the round pen with the aid of the lead. The animals were then released from the lead and kept loose in the round pen, where they were led to perform circular movements while maintaining the same principles of direction and control. Later, the trainer gave a vocal command to approach, initiating movement at a walk to stimulate voluntary following. This stage allowed for the evaluation of the animal’s responsiveness to the trainer’s stimuli and its ability to pay attention and maintain proximity at liberty. During this phase, the animals trained their walk, trot, and gallop, both with a lead and at liberty. The phase lasted an average of 30 days, with four sessions per week, each performed individually and lasting approximately 30 min per animal. Continuous HR, post-exercise ocular IRT, and post-exercise plasma cortisol were collected in a covered round pen immediately after the sessions. Baseline measurements (HR, ocular IRT, and cortisol) were taken before exercise, inside each animal’s stall.

- Phase 1—Steps:

Activities: Groundwork exercises conducted in a covered round pen without riding, including leading with a halter and long lead rope, circular locomotion at the walk, trot, and canter in both directions (five repetitions per direction), using pressure-and-release principles, vocal commands, body language cues, and training flags, followed by liberty work to encourage voluntary following and proximity to the trainer.

HR: Baseline resting heart rate measurements were obtained in the individual stall before exercise, followed by continuous heart rate monitoring throughout the taming session in the covered round pen.

Plasma Cortisol: Collection of a baseline resting blood sample in the individual stall before exercise, followed by a single post-exercise blood sample obtained immediately after the taming session in the covered round pen.

Ocular IRT: Baseline ocular IRT images were collected in the individual stall before exercise, followed by post-exercise image acquisition immediately after completion of the taming session in the covered round pen.

**Figure 2:**
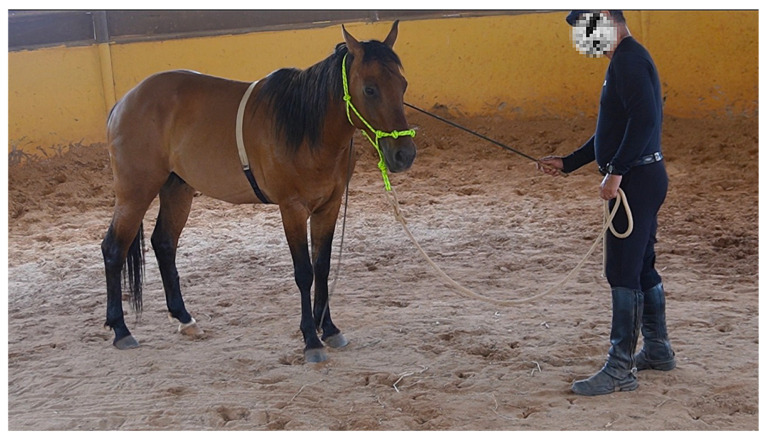
Desensitization process using a stick in a covered round pen. Use of a stick during the desensitization process conducted by the responsible trainer inside a covered round pen. In the image, the neck area is being desensitized. The Polar H10 sensor used for continuous heart rate monitoring is visible around the animal’s thoracic region.

Phase 2—Desensitization (30 days): Phase 2 aimed to desensitize the animals to external stimuli, reducing their reactivity to objects, sounds, and various forms of physical contact, as described in the literature for equine training protocols [38]. They were initially handled in the stall with the halter in place, then led to the round pen at a walk using a long lead. Upon reaching the round pen, the halter was removed, and the animals were encouraged to move freely and, for a short period, execute the basic commands previously learned in Phase 1. The stop command was then given to replace the halter and begin the desensitization exercises. The equipment included a halter, long lead, stick, flag stick, vocal commands, and the trainer’s body language. The desensitization process was conducted progressively. Initially, tactile stimulation was performed with a stick on different regions of the animal’s body, following a standardized sequence: head, neck, trunk, back, loin, rump, and limbs (Figure 2).

**Figure 3:**
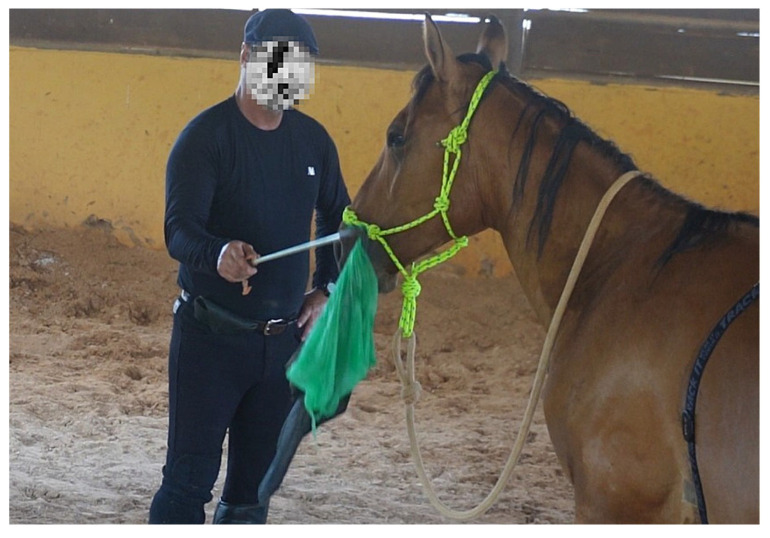
Desensitization process using a flag stick in a covered round pen. Use of the flag stick in the desensitization process conducted by the responsible trainer inside a covered round pen. In the image, the flag stick is being passed over the animal’s head. Note: The Polar H10 sensor used for continuous heart rate monitoring can be observed around the animal’s thoracic region.

Then, the same procedure was repeated with the trainer’s own hands to promote gradual adaptation to direct physical contact, always bilaterally. Following this, visual and auditory stimuli were introduced. Using the stick, movements were made around and above the animal without direct contact, as well as movements that generated ground vibrations, producing additional auditory stimuli. The use of the flag stick allowed for the introduction of more intense visual stimuli through approach and retreat movements across different body regions, including areas of greater and lesser sensitivity (Figure 3).

These stimuli were intended to increase the animal’s tolerance to situations commonly encountered in hippotherapy. They were initially presented while the animal remained stationary (standing still). Progression to application during locomotion (walk, trot, and canter) occurred when the horse was under the trainer’s control and could complete the exercise without persistent avoidance behaviors that would prevent the continuation of the session. The same progression sequence was applied to all animals, although advancement depended on each horse’s individual response. The progression of stimuli followed the same predefined order for all animals, moving from tactile to visual and auditory stimuli. However, progression between exercises depended on each horse’s ability to complete the previous task while maintaining responsiveness to the trainer’s verbal and body language cues. The saddle was introduced to the animals at the end of the 15th day of the month, progressively, until they allowed it to be placed on their backs and the girth tightened. As is common in Western saddles in Brazil, a girth and belly band (two girths) were used during desensitization. Taming continued until the animals could be calm at a walk and trot in a circle on the lead with the saddle. This phase lasted 30 days, with sessions held three times a week, lasting approximately 30 min per animal. Continuous HR, post-exercise ocular IRT, and post-exercise plasma cortisol measurements were taken in a covered round pen immediately after the sessions. Baseline measurements (HR, ocular IRT, and cortisol) were taken before exercise, inside each animal’s stall.

- Phase 2—Steps:

Activities: Progressive desensitization exercises were conducted in a covered round pen, including responses to previously learned groundwork commands, tactile stimulation with a stick and the trainer’s hands across different body regions, exposure to visual and auditory stimuli via stick and training flag movements, and gradual habituation to saddle placement and girthing. Stimuli were initially applied while stationary and subsequently during locomotion at the walk, trot, and canter, based on each animal’s acceptance.

HR: Baseline resting heart rate measurements were obtained in the individual stall before exercise, followed by continuous heart rate monitoring throughout the taming session in the covered round pen.

Plasma Cortisol: A baseline resting blood sample was collected in the individual stall before exercise, followed by a single post-exercise blood sample obtained immediately after the completion of the taming session in the covered round pen.

Ocular IRT: Baseline ocular IRT images were collected in the individual stall before exercise, followed by post-exercise image acquisition immediately after the completion of the taming session in the covered round pen.

Phase 3—Capacity Building and Skills Development (60 days): Phase 3 corresponded to the taming and qualification of the animals for use in hippotherapy sessions. The timeline for this phase is presented in Table 1.

In this stage, the animals were introduced to forward, sideways, circular, stopping, and backing movements, gradually progressing in the exploration of gaits. Everyday situations, objects, and experiences that typically occur in hippotherapy sessions were also presented. The materials were a halter, a lead rope, a complete bridle, a blanket, and a complete saddle. Taming began with progressive activities, adapting to the riding equipment, exposure to environmental stimuli, and the simulation of routine care situations to promote behavioral safety, emotional stability, and appropriate responses to the handler’s commands. During the materials presentation phase, the handler entered the round pen or arena and temporarily assumed a role in the taming process, sometimes leading the animal and assisting with taming, including desensitization to the saddle, bridle, and other equipment, until the first ride. After this stage, when the animal was responsive to the mounted trainer’s commands at the walk and trot, specific hippotherapy objects were introduced. Galloping was practiced during gymnastics taming. The materials included a halter, lead rope, complete bridle, blanket, and complete saddle. In this PTPH stage, the animals were led through a desensitization activity circuit with the stimuli described below, where the objects were left randomly or presented by a person known or unknown to the horse. The adaptation sequence to the objects was performed continuously, in a sequential circuit format within the location where they performed their tasks—in this case, a 30 × 40 m covered arena with a nonwoven fabric floor covered with sand, simulating a real hippotherapy session as it occurs in Brazil. The materials described below were presented sequentially during the taming sessions, following the same general order for all animals. Depending on the individual taming session, some stimuli could be repeated when additional habituation was required before progressing to the next exercise.

Cards and velcro: A deck of colored cards was presented and brought close to the animal, followed by tactile (touches on the neck) and auditory stimuli (opening and closing a bag with Velcro); finally, the cards and Velcro were moved over the animal’s back and loin.

Clipboard with sheets: Moving the clipboard and sheets near the animal’s head and then throwing the clipboard to the ground.

Plastic hoops: Four large, colored plastic hoops were arranged in a straight line on the ground so that, when the horse was led, it would pass through them without showing any refusal or jumping behavior.

Exercise ball: This was positioned on the ground of the arena, allowing the animal’s spontaneous interaction, such as sniffing, biting, or avoiding the object, according to its behavioral response.

One or more unknown people interacting from the ground:

Gentle touch: The person slowly touched the horse on previously desensitized areas.

Intense touch: The person touched the horse more firmly on the same areas.

Verbal interaction: The person established verbal communication with the animal, using constant intonation and altering vocalization.

Sudden vocal stimulus: After verbal interaction, the person emitted a high-pitched vocal sound near the animal to assess its reaction to an unexpected sound.

Soccer ball: This was initially presented for the animal to smell, and later used for desensitization through direct contact with the horse’s body, rolling the ball along the body surface. Then, the object was thrown or rolled over and under the animal, and its behavioral reactions were observed.

**Figure 4:**
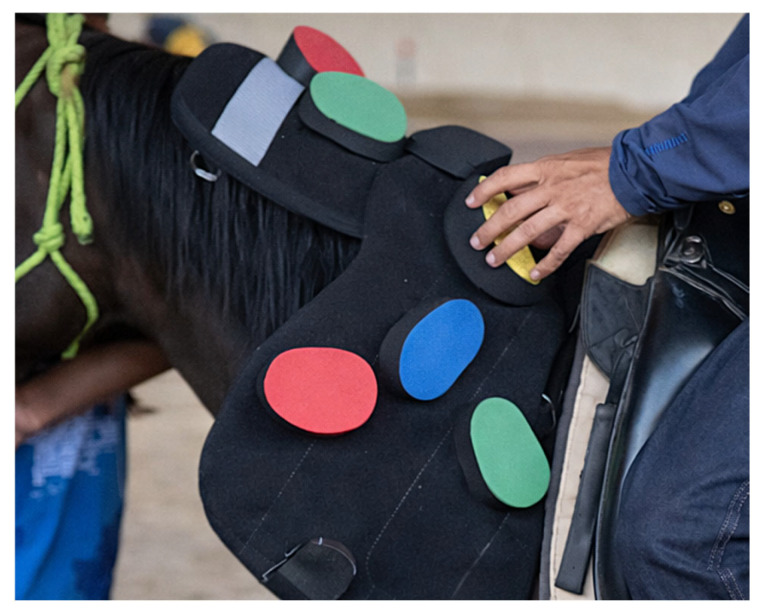
Miniature saddle with colorful geometric figures. Adapted equipment (a miniature saddle) is used for cognitive and motor stimulation in hippotherapy sessions. Colorful, textured geometric shapes, attached to the horse’s back with Velcro, serve as visual and tactile stimuli for practice during the therapeutic activity.

Miniature saddle with figures: This equipment was similar to a racing saddle with colored geometric figures attached by Velcro (Figure 4).

The figures were initially removed and repositioned in front of the animal; then, the equipment was positioned and slid along its back, repeating the same procedure (tactile and auditory stimulation).

Mirror: The trainer positioned the horse in front of a full-length mirror, allowing for the free observation of its behavioral reactions, such as vocalizations and behavior, intervening only in the case of attack.

Rings: Four colored rings were positioned on the animal’s face and then placed on the ears (two on each ear), while the trainer led the horse in a circle to the right and left.

**Figure 5:**
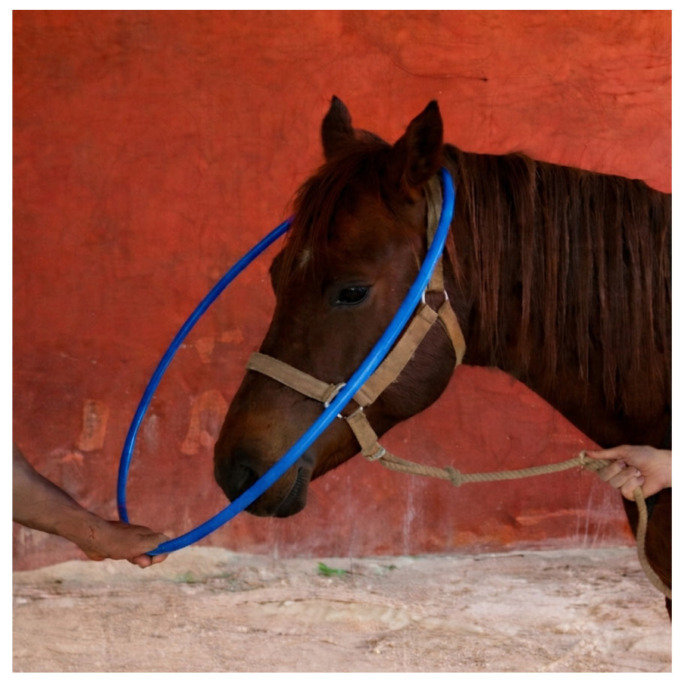
A plastic hoop is being passed around the head area of a horse during taming, as part of the desensitization and habituation process of the therapy horse. Placing the hoop around the animal’s neck demonstrates its docility, tolerance to unusual tactile and visual stimuli, and overall safety for hippotherapy activities.

Plastic hoop: A plastic hoop was carried along the animal’s body and inserted around the neck, while the horse was led by the halter, making a complete turn to both sides before the plastic hoop was removed (Figure 5).

Auditory stimuli using a sound device (speaker):

Counting: Playback of a recording containing numerical counting from one to five.

Days of the week: Playback of a recording with the names of the days of the week.

Sudden vocal stimulus: Playback of a recording containing the sound of a child screaming.

The last evaluation simulated a hippotherapy session under the conditions described above. The animal was initially saddled and warmed up on the lead, moving to the right and left sides, at a walk and trot, completing five full turns to each side. Then, each horse, in sequence, was ridden by the same trainer and led on the left side by the ground handler (acting as the assistant guide), performing a circuit (not timed) and going through the obstacles arranged in sequence (presented to the horse by an assistant) inside the arena. The last data collection occurred as soon as the horse entered the arena, and the final data collection occurred as soon as it finished the circuit. Continuous HR measurements, post-exercise plasma cortisol, and post-exercise IRT analysis were all performed in the covered arena during this phase. The first was performed at the end of the first 30 days, and the second at the end of the experiment (the last 30 days)—i.e., in February and at the end of March. Baseline HR, baseline IRT, and baseline cortisol were measured inside each animal’s stall. This phase lasted approximately 60 days, with an average frequency of four sessions per week and an average duration of 40 min per animal, which varied depending on individual response.

- Phase 3—Steps:

Activities: Mounted training and qualification for hippotherapy, including adaptation to riding equipment (saddle and bridle), the development of locomotor skills (forward, lateral, circular, halt, and rein-back movements), exposure to environmental, visual, tactile, and auditory stimuli commonly encountered during hippotherapy sessions, and progressive habituation to therapeutic objects, unfamiliar people, and simulated treatment scenarios. Training concluded with a standardized hippotherapy session simulation in a covered arena.

HR: Baseline resting heart rate measurements were obtained in the individual stall before exercise, followed by continuous heart rate monitoring throughout the taming and hippotherapy session simulations.

Plasma Cortisol: A baseline resting blood sample was collected in the individual stall before exercise, followed by a single post-exercise blood sample collected immediately after the completion of the taming or hippotherapy session simulation.

Ocular IRT: Baseline ocular IRT images were collected in the individual stall before exercise, followed by post-exercise images immediately after the completion of the taming or hippotherapy session simulation.

**Figure 6:**
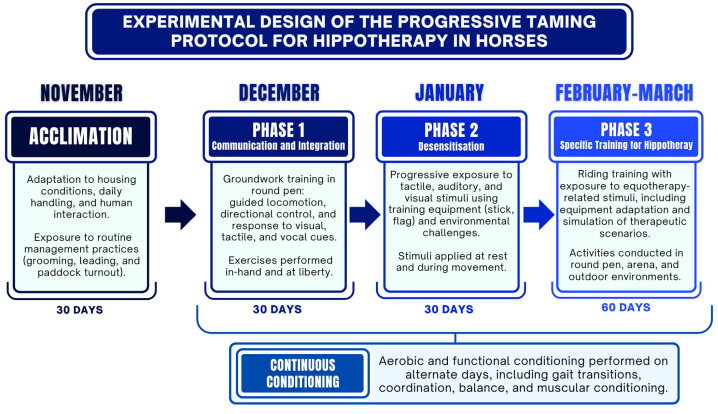
Visual diagram of the Progressive Taming Protocol for Hippotherapy (PTPH). Presentation of the respective months and phases of the Progressive Taming Protocol for Hippotherapy, encompassing the activities performed in each taming phase.

Gymnastics—physical conditioning, functional gymnastics, and/or basic training: The gymnastics phase consisted of continuous exercises performed throughout Phases 1, 2, and 3, lasting 30 min on alternate days, in addition to the other training activities. Its objective was to promote and maintain general physical conditioning, encompassing muscle strength, balance, motor coordination, and endurance, while specific hippotherapy training and taming were being established. These activities involved work on a lead or at liberty (in Phases 1 and 2 and part of the first 30 days of Phase 3) and outdoor or track work with basic training movements—e.g., 20–15–10–8 m circles, half turns, reverse half turns, serpentine turns, gallop starts, backing up, shoulder forward, leg yield (the latter only at the end of the last month), transitions in different gaits (walk, trot, gallop), small jumps (60–80 cm), poles, and barrels—also only in Phase 3. The exercises were performed for 30 min, on alternate days, with the taming days of each specific phase—except Sunday, when no animal was trained; all had this entire day to rest, loose in paddocks. The PTPH used in the study is summarized in Figure 6.

Statistical analysis: The data were analyzed using a linear mixed-effects model, considering month as a fixed effect and horse as a random effect. Normality and homoscedasticity were tested using the Shapiro–Wilk/QQ plot and Bartlett tests, respectively. The effect size was estimated using the Hedges method. Tukey’s test was used for multiple comparisons of means. The results were expressed as means and standard deviations, and *p*-values < 0.05 were considered significant. The R software (version 4.6.1) [39] was utilized for statistical analyses.

Linear Mixed Model (LMM):

Yij = μ + Mi + Cj + εij

where

Yij = observed heart rate;
μ = overall mean;
Mi = fixed effect of month;
Cj = random effect of horse;
εij = residual error.

Additional data supporting the findings of this study are provided in the Supplementary Materials (Supplementary File S1).

## 3. Results

The results are presented in the following sessions: HR, ocular IRT, and plasma cortisol analysis.

### 3.1. Heart Rate

Table 2 shows the variables analyzed and their respective measurement times. HR data were collected in two different locations: the first inside the stables and the second inside a covered arena.

HR_0_ differed between the months evaluated in the experiment (*p* = 0.0466). The greatest increase (40.57 ± 4.42 bpm) occurred in November, the month when the animals were adapting to the new environment. Baseline behavior remained within the expected normality over time. The variations are described in Table 3.

The highest HR_Min_ values were observed in December and January, corresponding to Phases 1 and 2 of the protocol, respectively. The values decreased throughout February and into March, when the lowest value was recorded (30.57 ± 2.76 bpm) (Table 4). The difference between December and March demonstrates the animals’ habituation to the taming protocol.

As for HR_Ave_ values, no differences were found (*p* = 0.168) between months (Table 5), indicating that the work performed during the protocol stages did little to alter the physiological cardiac patterns commonly observed in healthy animals performing similar exercises under similar conditions.

The highest HR_max_ value was observed in December (120.00 ± 41.98 bpm), while the lowest value was recorded in February (69.00 ± 20.85 bpm). The months of January and March showed intermediate values, not differing from the other months (Table 6). Taming began in December, after the adaptation period, which helps explain the greater increase in HR during this period. Small changes occurred in the other months; however, even with the expected adaptation to taming, which was apparently more present in February, an increase occurred in March, probably due to the characteristics of the exercise performed during that period.

HR_Fin_ values had no noteworthy difference between the months evaluated in the experiment (*p* = 0.0941) (Table 7), possibly indicating the ease and tendency of the animals to adapt and become accustomed to this method. HRs returned to physiological values immediately after the end of the exercise, and thus did not require longer monitoring periods.

HR variables differed from each other throughout the experiment. HR_max_ had the highest value (96.85 ± 37.89 bpm), differing from the other variables. HR_Ave_ had an intermediate value (54.92 ± 15.16 bpm), while HR_Fin_, HR_Min_, and HR_0_ did not differ from each other (Table 8). HR_Max_ ranged from 134.74 to 58.96 bpm. The large heartbeat amplitude is due not only to the variation in gaits (walk, trot, and gallop) but mainly to the variation in the animals’ perception of situations and objects presented at different times. HR_Max_ values likely reflected a combination of exercise intensity, exposure to novel stimuli, anticipatory responses, and transient autonomic arousal during the taming sessions. Therefore, these peaks should not be interpreted solely as indicators of stress. Also, HR_Ave_ ranged from 70.08 to 39.76 bpm, classifying the Progressive Taming Protocol for Hippotherapy as a light-intensity exercise. The similar HR_Fin_ (obtained immediately after the exercise in all stages), HR_Min_, and HR_0_ values suggest rapid cardiovascular recovery following exercise and may indicate good adaptation to the workload imposed during taming.

### 3.2. Ocular Infrared Thermography

#### Lacrimal Caruncle

The lacrimal caruncle region showed a difference in eye temperature between the months evaluated (*p* = 0.002). The highest temperature occurred in December (36.10 ± 0.64 °C), likely reflecting physiological stress associated with the onset of taming, combined with the elevated ambient temperatures typical of summer. It is believed that the work was well tolerated due to the observed decrease in lacrimal caruncle temperature in March (33.82 ± 1.17 °C) (Table 9). However, this reduction cannot be attributed solely to habituation; a significant portion of this variation is linked to seasonal differences in environmental conditions, as ocular surface temperature is highly susceptible to ambient climate fluctuations.

Eye temperature increased after exercise (37.01 ± 0.65 °C) in the first month of taming (December), as expected for the initial phase of taming exposure (Table 10). This did not recur in the following months, which can be interpreted as the animals’ adaptation to the proposed exercises. However, the IRT increased again immediately after exercise in the last month of the protocol, coinciding with the phase of greatest taming and gymnastics intensity. This may be consistent with the greater demands of the final taming exercises.

### 3.3. Plasma Cortisol

Plasma cortisol concentration remained stable throughout the experiment (*p* = 0.1133). This suggests that the protocol did not trigger a severe acute endocrine stress response in the horses evaluated (Table 11), indicating a potential neuroendocrine homeostasis during the evaluated period.

Plasma cortisol concentrations before and after the taming process between December 2024 and March 2025 also did not show relevant changes (Table 12).

## 4. Discussion

Based on HR, ocular IRT, and plasma cortisol assessment results, this study indicates that PTPH promoted physiological adaptation in the evaluated parameters, suggesting good adaptability to hippotherapy-oriented taming. HR parameters showed a tendency toward reduced autonomic activation over time, with a progressive decrease in baseline values and minimum HR, and adequate post-exercise recovery, suggesting progressive physiological adaptation to the taming routine [19,40,41]. Complementarily, ocular IRT demonstrated an initial increase in lacrimal caruncle temperature at the start of taming, followed by stabilization over the months; this indicates reduced reactivity to aversive stimuli and subsequent adaptation to the handling and training routine [32,42]. In parallel, plasma cortisol concentration remained stable throughout the period, with no differences between before and after exercise, suggesting that the intensity and nature of the activities were insufficient to trigger an acute endocrine stress response, consistent with the training protocol being well tolerated by the horses [43]. Taken together, these factors indicate that the PTPH protocol allowed for the controlled progression of physical and cognitive conditions consistent with the type of taming used for the indicated learning phases of this group of horses, favoring their habituation, learning, and physiological adaptation to new stimuli, without evidence of the impairment of their well-being.

HR: During the adaptation phase, HR_0_ values ranged from 33.71 to 40.57 bpm, remaining within the physiological range described for equines at rest (20–40 bpm) [44]. The highest value observed at the beginning of the experimental period may be associated with the animals’ introduction to a new environment, a condition that tends to increase alertness and autonomic activity [28,45]. Greater cardiovascular activation in the animals was observed in December (corresponding to Phase 1), characterized by increases in HR_Max_ and HR_Min_. This period was marked by the introduction of the first aversive taming stimuli, including floor work, desensitization, and exposure to objects, configuring a scenario of high novelty and emotional and cognitive demand.

The progressive modulation of cardiovascular responses was observed from January onwards, with the start of Phase 2, reflecting the animals’ adaptation to the taming protocol. The reduction in HR_Min_ values over time indicates a decrease in anticipatory sympathetic activation, associated with greater predictability of activities and with familiarization to the environment, handling, and stimuli [11,19,40,46].

The progressive reduction in HR_0_ over subsequent months suggests an adaptation process for handling [19,40,47], possibly related to familiarization with the routine, the environment, and interactions with the handler and trainer [19]. This process is associated with the modulation of the autonomic nervous system, with a predominance of parasympathetic tone over time, reducing resting HR [48,49,50]. The close HR_Fin_, HR_Min_, and HR_0_ values throughout this phase indicate a consistent pattern of physiological stability, suggesting adequate autonomic regulation even in the face of progressively increasing taming complexity [28,51]. This behavior indicates that, in addition to post-exercise recovery capacity, the animals maintained a balance between the sympathetic and parasympathetic systems throughout the sessions [49,52].

The wide variation observed in HR_Max_ can be attributed not only to taming in different gaits but mainly to the variability of the stimuli presented during taming. This includes the introduction of new objects, changes in the environment, and new situations, which directly influence the animals’ perception and reactivity [11,40,45]. The reduction in HR_Max_ in February, with the lowest value recorded throughout the experiment, indicates a period of greater adaptation to the physical demands of taming. This behavior is consistent with reduced reactivity to previously presented stimuli [40,41,53]. In contrast, the increase in HR_Max_ in March may be related to the introduction of more complex stimuli, such as taming, new objects, and circuits with multiple simultaneous stimuli, characteristic of this more advanced phase of taming, which combines greater physical and cognitive demands [17,40,45,51,54]. A higher HR_Max_ (120 bpm) indicates a marked sympathetic response to new or potentially challenging stimuli [28,45,49,51,52,55,56], while a higher HR_Min_ suggests increased anticipatory activation at the start of exercise, possibly associated with anticipation of the proposed activities [45,57]. There were specific moments when HR_Max_ was unexpectedly high, which we termed “peak” rates. This occurred when a horse sighted an unfamiliar object that caused fright or fear until habituation was complete. This explains some of the highest HR_Max_ values in December and March, particularly.

Despite this increase, HR_Ave_ remained within a stable pattern, possibly because it was not a direct response to workload but rather to the direct exposure of the horses to new stimuli, such as unfamiliar objects or different forms of handling, which can increase HR_Ave_ as a result of emotional excitement and response to stimuli [43,44,45]. This result suggests that the changes observed in the other HR variables are more related to the animals’ response to stimuli than to the workload itself [30,41]. The maintenance of HR_Ave_ without significant differences across months reinforces that the physiological load of the exercise remained relatively constant throughout the experimental period, even with increased activity complexity, suggesting that the protocol was conducted in a progressive and controlled manner [30,41,53]. Horses undergoing progressive taming are expected to show improved aerobic capacity (assessed by the kinetics of post-exercise HR recovery and the shorter time required to return to baseline) and greater efficiency of HR recovery after exertion, reflecting cardiovascular adaptation and better autonomic balance [57,58,59]. However, the evaluated protocol did not have sufficient physical intensity to induce such chronic adaptations of athletic training. On the other hand, the lack of difference between HRFin and HR0 demonstrates that the animals finished the session in a state of cardiovascular homeostasis, a response found in horses subjected to light gait and low-impact activities, such as hippotherapy [29], where the return and maintenance of HR below the threshold of 60 bpm at the end of the activity (41.03 ± 6.36 bpm) indicate that the effort did not generate overload, stress, or fatigue in the animals [30], justifying the rapid recovery observed.

Ocular IRT: Because this is not a conventional measure, some data must be described: the lacrimal caruncle was the preferred anatomical region, as measurements of stress levels in horses via ocular IRT demonstrated a high capacity for vasodilation in this location, favoring heat dissipation and making the measurement more accurate [34,60,61,62]. Physiologically, this highly vascularized region responds to autonomic activation, translating stress into rapid shifts in ocular temperature [63]. Furthermore, its accessibility allows non-invasive imaging, minimizing handling-induced stress [60]. Moreover, the 0.5 m capture distance demonstrated less variability and less influence from external factors in the measurements, as expanding the camera’s field of view may include adjacent tissues and distort the average values [31,34,64]. Thus, the use of 0.5 m favors greater accuracy and reproducibility of thermographic measurements [34].

Although the adaptation phase is considered stressful, the values obtained in November were considered normal for baseline (34.40 ± 0.60 °C) [31,42,65], as changes in physiological parameters associated with alertness and autonomic activation are typically expected [28,45]. Nonetheless, maintaining eye temperature within the physiological range suggests the absence of significant changes in baseline peripheral perfusion, indicating that the animals’ physiological response to the new environment may not have been sufficient to produce detectable changes in ocular IRT [42,65].

However, the comparison of values obtained in November (34.40 °C) and December (36.10 °C) shows a significant increase in the pre-exercise temperature of the lacrimal caruncle, indicating greater physiological activation of the animals during this period. This increase coincides with the beginning of the taming process (Phase 1), in which the animals were led to the covered round pen and exposed, for the first time, to structured handling and taming activities, including tactile, visual, and auditory stimuli not previously consolidated, characterizing a scenario of novelty and potential challenge [11]. In this context, the temperature of the lacrimal caruncle in horses can increase in response to acute stimuli, reflecting the activation of the sympathetic nervous system and changes in peripheral blood flow [32,42,60,66]. The magnitude of this baseline (at rest inside the stable) increase may be associated with the multifactorial nature of the stimuli applied in this phase, which involved simultaneous physical, sensory, and emotional components, potentiating the physiological response. Thus, such an increase may be related to the animals’ greater reactivity to taming stimuli, rather than necessarily to negative stress, and is compatible with excitation and adaptation responses to the environment [40,53].

A marked reduction in the temperature of the lacrimal caruncle was observed in January (33 °C) in relation to December (36.10 °C). This behavior can be attributed to the adaptability of horses, which have a high aptitude for learning when subjected to adequate management and taming conditions, favoring the development of habituation processes [11].

The thermal values observed in February were similar to those in January, with no significant increases, suggesting the maintenance of the animals’ thermal homeostasis through adaptive thermoregulation in response to PTPH stimuli. Physiologically, physical training and repetitive handling improve the efficiency of heat dissipation (sweating), thereby preventing excessive heat storage and helping to stabilize internal temperature under the same workload [41,67]. This adjustment strengthens the hypothesis that the adaptive physiological response is stabilized throughout the experimental period [29,57,68]. This pattern suggests that the controlled repetition of stimuli promoted the modulation of autonomic activity and reduced reactivity to stress, a result consistent with findings describing a gradual decrease in eye temperature throughout the training period in horses [42,69]. Physiologically, this progressive reduction and stabilization of the baseline ocular temperature over months reflect decreased anticipatory sympathetic tone and improved thermoregulatory efficiency [41,42,69], suggesting that eye temperature may serve as an indicator of physiological adaptation to a continuous taming protocol.

Regarding the moments before and after exercise in January and February (Phases 2 and 3), the controlled repetition of stimuli, combined with adequate taming conditions, favored the modulation of sympathetic activity and the reestablishment of autonomic balance. This led to the stabilization of peripheral microcirculation and, consequently, the absence of differences between these moments [11,32,66,69], indicating physiological adaptation to the taming environment, with a reduction in stress reactivity.

Considering baseline values, no increase in lacrimal caruncle temperature was observed in March compared to previous months. This behavior reinforces the habituation process and indicates consistent physiological adaptation to handling and taming throughout the experimental period [66]. Altogether, the analysis of baseline monthly variations shows a progressive reduction in physiological reactivity to repeated stimuli, characterizing the horses’ adequate adaptive response to the taming process [69].

However, post-exercise values increased in March during Phase 3 (36.41 ± 0.44 °C), unlike in December, which may be more related to the physiological demands of the final phase of taming than to a psychological stress response. In this stage, the animals were subjected to riding and other more complex activities, which may have increased muscle activity and metabolic rate, resulting in greater heat production and activation of peripheral thermoregulation mechanisms [70,71].

Plasma cortisol: There was no difference throughout the months or before and after the exercise tests. This suggests that the intensity and duration of the proposed activities were not sufficient to trigger an acute endocrine response, as in the HR and ocular IRT evaluation, possibly because the magnitude of the cortisol response in horses is strongly dependent on the intensity, duration, and nature of the exercise, being more evident in protocols of high metabolic demand or in situations of intense physical stress [72,73,74,75,76,77], which probably did not occur during the taming proposed in this study. PTPH, structured around gradual exposure to taming stimuli, was associated with stable physiological responses throughout the experimental period. Together, the HR, ocular IRT, and plasma cortisol findings suggest progressive adaptation to the taming process, with no evidence of marked physiological disruption.

An assessment of the influence of aerobic taming on plasma cortisol and glucose concentrations in untrained horses subjected to a physical conditioning program [43] also found no differences in baseline values at rest after the taming period, indicating a physiological adaptation to exercise through modulation of the hypothalamic–pituitary–adrenal axis that reduces the magnitude of the endocrine response to previously stressful stimuli, regardless of the exercise type.

The convergence between the indicators evaluated in this study—heart rate, ocular IRT, and plasma cortisol—reinforces that the controlled progression of activities in PTPH favored habituation to stimuli and the modulation of autonomic responses over time, suggesting that the protocol may represent a potentially suitable approach for preparing horses for hippotherapy.

Study limitations: This study was unable to incorporate multiple complementary behavioral parameters, including standardized behavioral scoring systems and formal welfare assessment tools, which could have provided additional insight into the horses’ responses throughout the taming process. Additionally, due to the specific field conditions of the taming protocol, increasing the sample size or including a control group undergoing an alternative taming modality for comparison was not feasible. Furthermore, baseline standardized characterization of exercise intensity (such as treadmill tests or combined field-effort assessments using HR and lactate profiling) was not performed to quantify the intensity of this type of exercise taming in QHs prior to this study. Finally, although plasma cortisol concentrations may exhibit delayed peaks following stress or exercise exposure, serial post-exercise sampling at multiple time points was not performed.

## 5. Conclusions

Taken together, the HR, cortisol, and ocular IRT results indicate that PTPH promotes gradual physiological adaptation in horses across the evaluated parameters, supporting physiological adaptation to the taming process used in equine therapy. This type of protocol (PTPH), directed toward the horse’s final activity, respects the animal’s adaptive and physiological capacity. These findings may provide a basis for future studies aimed at optimizing horse preparation protocols for equine-assisted interventions.

## Figures and Tables

**Table 1:** Temporal organization of Phase 3.

Taming Period	Duration	Weekly Frequency	Location
Beginning	First 30 days	4 times a week	Covered round pen (twice a week), outdoor track (once a week), and covered arena (once a week)
End	Last 30 days	4 times a week	Outdoor track or covered arena, alternating as needed for training

Taming period, including duration, weekly frequency, and location.

**Table 2:** Variables analyzed and their respective measurement times during heart rate tests.

Variable	Measurement Time
Baseline Heart Rate (HR_0_)	Measurement inside the stables, before the start of taming
Minimum Heart Rate (HR**_Min_**)	Minimum value recorded during taming in the covered arena
Average Heart Rate (HR**_Ave_**)	Average value recorded throughout the taming session in the covered arena
Maximum Heart Rate (HR**_Max_**)	Maximum value recorded during taming in the covered arena
Final Heart Rate (HR**_Fin_**)	Measured immediately after the end of taming in the covered arena

**Table 3:** Average HR_0_ values collected between November 2024 and March 2025.

Months	Mean (bpm) ± SD	*p*	Hedges’ g
November	40.57 ± 4.42 ^a^	0.0466	1.70
December	37.14 ± 3.23 ^ab^		
January	35.42 ± 4.27 ^ab^		
February	33.71 ± 2.92 ^b^		
March	34.85 ± 5.52 ^ab^		

Averages followed by different letters in the column differ from each other by Tukey’s test (*p* < 0.05).

**Table 4:** Average HR_Min_ values collected between December 2024 and March 2025.

Months	Adjusted Average (bpm) ± SD	*p*	Hedges’ g
December	39.71 ± 4.07 ^a^	0.0095	2.45
January	37.71 ± 6.02 ^a^		
February	35.85 ± 4.41 ^ab^		
March	30.57 ± 2.76 ^b^		

Averages followed by different letters in the column differ from each other by Tukey’s test (*p* < 0.05).

**Table 5:** Average HR_Ave_ values collected between December 2024 and March 2025.

Months	Adjusted Average (bpm) ± SD	*p*	Hedges’ g
December	63.28 ± 19.23 ^a^	0.168	1.01
January	55.00 ± 15.23 ^a^		
February	46.57 ± 10.33 ^a^		
March	54.85 ± 12.80 ^a^		

Averages followed by different letters in the column differ from each other by Tukey’s test (*p* < 0.05).

**Table 6:** Average HR_max_ values collected between December 2024 and March 2025.

Months	Adjusted Average (bpm) ± SD	*p*	Hedges’ g
December	120.00 ± 41.98 ^a^	0.0272	1.44
January	91.57 ± 33.95 ^ab^		
February	69.00 ± 20.85 ^b^		
March	106.86 ± 37.84 ^ab^		

Averages followed by different letters in the column differ from each other by Tukey’s test (*p* < 0.05).

**Table 7:** Average HR_Fin_ values collected between December 2024 and March 2025.

Months	Adjusted Average (bpm) ± SD	*p*	Hedges’ g
December	43.14 ± 6.09 ^a^	0.0941	1.08
January	44.57 ± 7.21 ^a^		
February	38.71 ± 6.10 ^a^		
March	37.71 ± 4.15 ^a^		

Averages followed by different letters in the column differ from each other by Tukey’s test (*p* < 0.05).

**Table 8:** Adjusted averages of heart rate variables were compared throughout the experiment (December 2024 to March 2025).

Heart Rate	Adjusted Average (bpm) ± SD	*p*	Hedges’ g
HR_Max_	96.85 ± 37.89 ^a^	<0.001	2.11
HR_Ave_	54.92 ± 15.16 ^b^		
HR_Fin_	41.03 ± 6.36 ^c^		
HR_Min_	35.96 ± 5.45 ^c^		
HR_0_	35.28 ± 4.08 ^c^		

Averages followed by different letters in the column differ from each other by Tukey’s test (*p* < 0.05).

**Table 9:** Average baseline ocular temperature (°C) of the animals’ lacrimal caruncle of the left eye between November 2024 and March 2025.

Months	Average (°C) ± SD	*p*	Hedges’ g
November	34.40 ± 0.60 ^b^	0.002	2.25
December	36.10 ± 0.64 ^a^		
January	33.92 ± 1.34 ^b^		
February	34.62 ± 0.68 ^ab^		
March	33.82 ± 1.17 ^b^		

Averages followed by different letters in the column differ from each other by Tukey’s test (*p* < 0.05).

**Table 10:** Average temperatures (°C) found in the lacrimal caruncle region from December 2024 to March 2025.

Months	Time Point		*p*	Hedges’ g
	Before	After		
December	36.10 ± 0.64 ^b^	37.01 ± 0.65 ^a^	0.0202	1.30
January	33.92 ± 1.34 ^a^	34.01 ± 1.89 ^a^	0.922	0.05
February	34.62 ± 0.68 ^a^	36.32 ± 2.28 ^a^	0.1062	0.94
March	33.82 ± 1.17 ^b^	36.41 ± 0.44 ^a^	0.002	2.73

Averages followed by different letters within the row differ significantly according to Tukey’s test (*p* < 0.05).

**Table 11:** Average baseline plasma cortisol values (ng/mL) measured between November 2024 and March 2025.

Months	Average (ng/mL) ± SD	*p*	Hedges’g
November	30.32 ± 9.88 ^a^	0.1133	1.50
December	23.02 ± 6.49 ^a^		
January	22.21 ± 6.43 ^a^		
February	22.47 ± 10.63 ^a^		
March	18.37 ± 3.41 ^a^		

Averages followed by the same letter in the column do not differ from each other by Tukey’s test (*p* < 0.05).

**Table 12:** Average plasma cortisol concentrations before and after taming between December 2024 and March 2025.

Months	Time Point		*p*	Hedges’g
	Before	After		
December	23.02 ± 6.49 ^a^	28.55 ± 8.50 ^a^	0.3191	0.68
January	22.21 ± 6.43 ^a^	23.34 ± 5.33 ^a^	0.7062	0.18
February	22.47 ± 10.63 ^a^	21.98 ± 6.62 ^a^	0.8835	0.05
March	18.37 ± 3.41 ^a^	19.70 ± 6.18 ^a^	0.4489	0.25

Averages followed by the same letter in the row do not differ from each other by Tukey’s test (*p* < 0.05).

## Data Availability

The original data presented in the study are publicly available at Protocols.io at DOI: https://dx.doi.org/10.17504/protocols.io.kxygxjjxkl8j/v1.

## Supplementary Materials

The following supporting information can be downloaded at: https://www.mdpi.com/article/10.3390/ani16131980/s1, File S1: Data Analysis.
